# Tissue-specific transcriptomic adaptation in three strains of chickens during coinfections with parasites

**DOI:** 10.1186/s13099-025-00716-1

**Published:** 2025-06-11

**Authors:** Oyekunle John Oladosu, Henry Reyer, Nares Trakooljul, Solvig Görs, Cornelia C. Metges, Gürbüz Daş

**Affiliations:** Research Institute for Farm Animal Biology (FBN), Wilhelm-Stahl-Allee 2, 18196 Dummerstorf, Germany

**Keywords:** Caecum, Immune response, Jejunum, Liver, Metabolic, Nematode, Protozoa, Resistance, Tolerance

## Abstract

**Background:**

The widespread adoption of non-caged production systems contributes to the pathogenesis of coinfections with gastrointestinal nematodes and *Histomonas meleagridis*, triggering local and systemic immune and metabolic responses in chickens. This study investigated transcriptomic adaptation of tissues two weeks after experimental coinfections with *Ascaridia galli, Heterakis gallinarum*, and *H. meleagridis* in Lohmann Brown (LB), Lohmann Dual (LD), and Ross-308 (R) male chickens, which differ in growth rates (R > LD > LB).

**Results:**

RNA sequencing of the jejunum, caecum, and liver tissues revealed tissue-specific, strain-dependent transcriptional responses. Coinfection was confirmed during necropsy, and Ascaris-specific antibodies as well as alpha-1-acid glycoprotein were significantly higher in infected birds (*p* < 0.01). The caecum exhibited the highest unique differentially expressed genes, DEGs (n = 4,094), corresponding to significant activation of complex immune pathways and inhibition of metabolic pathways (*p* < 0.01). Jejunum DEGs (n = 760) primarily related to muscle contraction, collagen metabolism notably in LB and LD strains. The liver displayed fewer unique DEGs (n = 266) but prominently activated immune responses, especially in R chickens.

**Conclusion:**

In general, slower-performing strains effectively initiated responses favouring worm expulsion and tissue repair in the jejunum, whereas high-performing strains predominantly showed inflammatory responses in the caecum and liver. These findings highlight tissue-specific adaptations underlying strain-dependent tolerance to coinfections with mixed parasites.

**Supplementary Information:**

The online version contains supplementary material available at 10.1186/s13099-025-00716-1.

## Background

Coinfections of helminths are a common occurrence in chickens, especially in those raised in outdoor production systems [19, 44, 48]. Studies have also demonstrated that helminth infections can significantly influence the pathogenesis of other infections and the host’s immune response to other pathogens, potentially exacerbating pathophysiological changes within the host [7, 28, 34]. The immune system becomes burdened and its ability to effectively respond to simultaneous infections can be compromised. Coinfections with the nematode species *Ascaridia galli* and *Heterakis gallinarum*, concurrently with the extracellular protozoan *Histomonas meleagridis*, are of particular interest due to their high prevalence and ease of transmission. Both *A. galli* and *H. gallinarum* are transmitted via the oral-faecal route in chickens [35], whereas *H. meleagridis* transmission occurs mainly through the ingestion of *H. gallinarum* eggs [17]*.* Transmission of *H. meleagridris* can however also occur via ingestion of intermediate mechanical hosts like earthworms, flies or other invertebrates carrying histomonad-infected eggs of *H. gallinarum*, or via direct bird to bird transmission [5, 12, 26, 30].

Helminth infections, regardless of species, typically induce a Th2-skewed immune response that is mediated by various immune components, including at least the activation of B cells and the stimulation of effector cytokines such as IL-4, IL-13, and IL-9 [4, 16]. In contrast, infections with *H. meleagridis* are characterized by an increased production of IFN-γ [23], which originates differently from the Th2 profile typically observed with nematodes. Notably, coinfection of *H. gallinarum* with *H. meleagridis* has been shown to induce a differential immune response compared to a *H. gallinarum* mono-infection [43]. Coinfected chickens also exhibit a more severe destruction of the caecal mucosa and significant T-cell infiltration in the caecal lamina propria [43], highlighting the complexity of immune response to coinfection. Additionally, *H. meleagridis* is transported via the hepatic vein to the liver, causing necrotic lesions and inducing further inflammatory responses [17]. This is particularly the case in turkeys, where a more lethal form of infection typically occurs [37]. Turkeys suffer fatal outcomes due to delayed innate immunity and dysregulated adaptive responses while chickens tolerate the infection via a more strong antibody response and balanced immunity [31, 37].

The pathological effects of parasite coinfections extend beyond immune modulation; they can also cause significant alterations in the host's metabolome [27]. Our previous study with laying hens also demonstrated alterations in host metabolism during concurrent nematodes and protozoan infections [32]. Infection often results in an imbalance in the host's ability to simultaneously initiate an effective immune response while maintaining an optimal metabolic state. Nutrient allocation is critical for the survival of an organism, and resources devoted to immune functions may come at the expense of resources for growth, reproduction, and other physiological processes [13, 40]. In chickens, peak immune responses to helminth infections corresponds with diminished laying performance in highly productive strains suggesting higher performing strains exhibit lower tolerance to coinfections [46]. Similarly, in high-performing broilers, the liver exhibited a prioritisation of immune-related biological processes over metabolic ones during infection [33]. In such strains, the intensified allocation of energy and nutrients for rapid growth, driven by genetic selection, limits their tolerance to environmental challenges [41].

Given the complexities of host-parasite interactions, we hypothesised that coinfection with *A. galli, H. gallinarum*, and *H. meleagridis*, each targeting distinct tissues and stimulate unique, site-specific pathological responses underlying differences in tolerance among chicken strains. Therefore, distinct transcriptomic signatures in the jejunum, caecum, and liver, that reflect their respective roles in parasite establishment and metabolic regulation, may reveal adaptive mechanisms unique to each strain. The jejunum is involved not only in local immune responses to intestinal pathogens like *A. galli* but also in nutrient absorption. The caecum serves as the primary infection site for *H. gallinarum* and the entry point for *H. meleagridis*, providing critical insights into early host defence mechanisms against protozoan invasion. Although the liver is not directly infected by nematodes, it becomes a secondary target for *H. meleagridis*, playing a significant role in systemic immune regulation and metabolism during infection. This study thus aimed to comprehensively characterise the transcriptomic responses at key predilection sites, i.e. jejunum, caecum, and liver, to understand the tolerance mechanisms of chickens to parasitic coinfection.

## Methods

### Ethics

The study was performed in compliance with established animal welfare regulations concerning animal care, handling, stunning, and necropsy procedures. Approval was obtained from the State Ethics Committee for Animal Experimentation (Mecklenburg-Western Pomerania State Office for Agriculture, Food Safety and Fishery, Germany; permission no: AZ: 7221.3–1–066/15). The procedures for experimental infection adhered to the guidelines by the World Association for the Advancement of Veterinary Parasitology [52].

### Experimental animals

Thirty-six (36) male chickens of the Lohmann Brown-LB, Lohmann Dual-LD, and Ross-308 strains (n = 12 per strain), aged one week, were used in this study. The three strains were selected for their different growth rates, which are expected to exert different levels of performance pressure that can make a strain more sensitive to environmental stressors, including pathogens. The birds were housed in pens of the experimental facility, where wood shavings were provided as litter material and left unchanged throughout the duration of the experiment. The chickens were fed ad libitum with a commercial diet containing 219 g/kg crude protein and 12.6 MJ/kg of metabolizable energy. No vaccinations, anthelmintics, or antiprotozoal treatments were administered to the birds during the study. Environmental conditions in the pens were regulated by an automated system to maintain consistent temperature, air flow, and lighting across all experimental rooms with initial temperature of 34 °C.

### Experimental design and infection model

Detailed descriptions of the experimental design can be found in previously published work, from which the subset of chickens (n = 36) used in this study was taken [47]. Briefly, chickens were randomly assigned to pens housing birds of the same strain. Each pen was further categorised to hold only infected or uninfected chickens of the same strain in a 3 × 2 factorial arrangement (three strains × two infection statuses). For biosecurity reasons, pens of the infected groups were located in a room separated from the uninfected groups to prevent cross-contamination between the two groups. The entire experiment was conducted in two temporal runs, and animals used in this study were from both temporal runs, each run comprising 18 chickens: 9 infected birds (n = 3 per strain) and 9 uninfected birds (n = 3 per strain), resulting in a total of 36 chickens across both runs. The infected group (aged 1 week) received an oral inoculation of 500 infectious eggs (in 0.2 mL saline solution) of *A. galli* and *H. gallinarum* while the control group was given an oral administration of saline solution (0.2 mL) also at one week of age. The parasite eggs were harvested from the intestines of naturally infected chickens. No direct administration of a defined dose of *H. meleagridis* was performed. The protozoan was introduced to the host via its main vector i.e. *H. gallinarum* eggs harboring *H. meleagridis*, which is known to be an efficient way to induce the infection as demonstrated with the presence of *H. meleagridis* in cecal tissue and lumen and along with histopathological lesions in a different cohort of the birds from the three host strains as reported earlier [11].

### Tissue sampling

At two weeks post-infection (wpi), the chickens from each run were stunned and killed by bleeding to assess the presence of parasites. The gastrointestinal tract was dissected longitudinally to confirm the infection status in the infected group and to verify the absence of parasites in the control group. Exact worm counts were not performed in these birds as the entire intestinal contents, including worms, were reserved for several different analyses. The gastrointestinal tract was separated, with the caecum and proximal jejunum isolated from the rest of the intestine. Liver samples were also collected. All tissue samples were immediately snap-frozen in liquid nitrogen, transferred to cryo-tubes and stored at −80 °C for subsequent analysis.

### Assessment of infections and host performance

The birds were weighed weekly in pens, and pen-based feed intake was also recorded. Blood samples were collected during necropsy at 2 wpi and placed in K-EDTA tubes (Sarstedt, Germany), which were then immediately placed on ice. Centrifugation of blood at 2500 g for 20 min at 4 °C was performed to separate the plasma and was subsequently stored at − 20 °C until further analysis. Anti-Ascaridia specific IgY levels in the EDTA-plasma samples were quantified using an Ascaridia-specific enzyme-linked immunosorbent assay (ELISA) [10]. The intra-assay coefficient of variability (CV) and inter-assay CV for this analysis were determined to be 5.0% and 8.4%, respectively. The acute-phase protein, alpha (1)-acid glycoprotein (AGP), was measured in plasma samples using a commercial ELISA kit, following the manufacturer's instructions (Life Diagnostics, West Chester, USA, Catalogue number: AGP-5).

For the analysis of free amino acids (AA), plasma samples collected during necropsy as described above was stored at −80 °C until analysis. Thawed plasma samples were prepared, and the AA concentration was measured using HPLC equipped with a fluorescence detector (Series 1200; Agilent Technologies, Waldbronn, Germany) and a 250 × 4 mm Hyperclone ODS (C18) 120 Å column, (Phenomenex, Aschaffenburg, Germany) following the method described in [22].

### RNA extraction, library preparation, and sequencing

Frozen tissue samples (~ 40 mg) from the jejunum, caecum, and liver (N = 108) were homogenized to extract total RNA, with subsequent steps of TRI reagent-based extraction (Sigma-Aldrich, Taufkirchen, Germany), DNase treatment (Roche Diagnostics, Mannheim, Germany) in solution, and RNA purification (NucleoSpin RNA kit; Macherey–Nagel, Düren, Germany) performed according to the manufacturer's instructions. One microgram of total RNA (RIN > 8) was utilized for library construction with the Illumina Stranded mRNA Prep, Ligation Kit, which included a 10-cycle PCR amplification as per the manufacturer’s guidelines (Illumina). The quality of the libraries was assessed using a Bioanalyzer to determine fragment length distribution, and the libraries were normalized to a concentration of 10 nM before pooling. Multiplexed DNA libraries were sequenced paired-end to generate 2 × 59 bp reads at a concentration of 750 pM on the NextSeq 2000 system, employing a P3 flow cell at the sequencing facility of the Research Institute for Farm Animal Biology (FBN), Dummerstorf, Germany. Raw sequencing reads in FASTQ format were produced using Dragen bcl convert v3.10.11 and subsequently assessed for quality with FastQC version 0.11.9 (http://www.bioinformatics.babraham.ac.uk/projects/fastqc/). Data preprocessing was conducted with Trim Galore v.0.6.10 (https://www.bioinformatics.babraham.ac.uk/projects/trim_galore/), during which low-quality reads (mean Q-score < 20) and short reads (< 20 bp) were discarded. Additionally, adapter-like sequences at the 3'-end of the sequence reads were trimmed. The remaining high-quality paired reads were aligned to the current chicken reference genome (GRCg7b) from Ensembl release 111 using Hisat2 version 2.2.1 [21]. The number of reads uniquely mapped to each gene was extracted from the aligned BAM file using HTSeq version 2.0.2 [38].

### Data analysis

Differential gene expression analysis was performed using the DESeq2 package in R [24]. The gene count data were pre-processed by removing low-expressed transcripts, retaining only those with at least 100 counts across a minimum of 5 samples. The Wald test was applied to determine differentially expressed genes (DEG) reaching the significance threshold of a false discovery rate (FDR) < 0.05. Contrasts of interest were generated for all pairwise comparisons between infection statues (i.e. infected vs control) and within each strain (i.e. LB, LD, R) across all tissues (i.e. jejunum, caeca and liver). Cook’s distance was used to identify transcript-level outliers. Partial least squares discriminant analysis (sPLS-DA) was performed using the mixOmics R package [42] to identify and rank the genes that best discriminate between the infection and strain groups. The model’s loadings were further examined to select the topmost influential genes used to generate a biplot. Genes were classified as significant if they met the criteria of FDR < 0.05 and were further analysed for gene ontology (GO) using the R/Bioconductor package topGO, focusing on Biological Process (BP) terms [2]. For each tissue, BP terms were analysed based on log_2_ fold change (log_2_FC) and FDR values (FDR < 0.05) of the corresponding genes. Activated BPs included genes with a positive log_2_FC and FDR < 0.05, while inhibited processes were enriched with genes with negative log_2_FC and FDR < 0.05. Enrichment analysis was conducted using Fisher’s exact test with the weighted algorithm (weight01) as implemented in the topGO package [2]. For each tissue, a topGOdata object was created with a minimum node size of 15, ensuring that only GO terms annotated with at least 15 genes were included. Following the enrichment test, raw *p*-values were adjusted for multiple testing using the Benjamini–Hochberg (BH) method and GO terms with adjusted *p*-values < 0.01 were considered significant. Additionally, a custom panel of genes involved in barrier function, nutrient transport, metabolism, oxidation, and immune response was selected from the literature [1, 6]. These genes were subsequently mapped to the differential expression results generated from our RNA-seq data in caecum, jejunum, and liver tissues to evaluate whether these genes were overrepresented in the differentially expressed gene sets. The statistical model for feed intake and growth performance, antibody levels against ascarids, and concentrations of plasma acute-phase protein (AGP) data included the fixed effects of infection, strain, and their interaction, adjusted for pen and run effects. The variables were analysed using the PROC GLM procedure of the SAS OnDemand for Academics cloud-based software (2021 SAS Institute Inc., Cary, NC, USA). Linear discriminant analysis (LDA) was performed using the MASS package in R [51] to assess the relationship between amino acid profiles, infection status, and genotype. The analysis aimed to identify amino acids that contributed most to the group separation. Additionally, a bar plot of amino acid loadings was used to highlight key discriminating amino acids for infection status. Two-sample t-tests were conducted to compare amino acid concentrations between the two infection groups. All visualizations were produced in R version 4.3.1 [39].

## Results

### Infection status and amino acid profiles

**Fig. 1:**
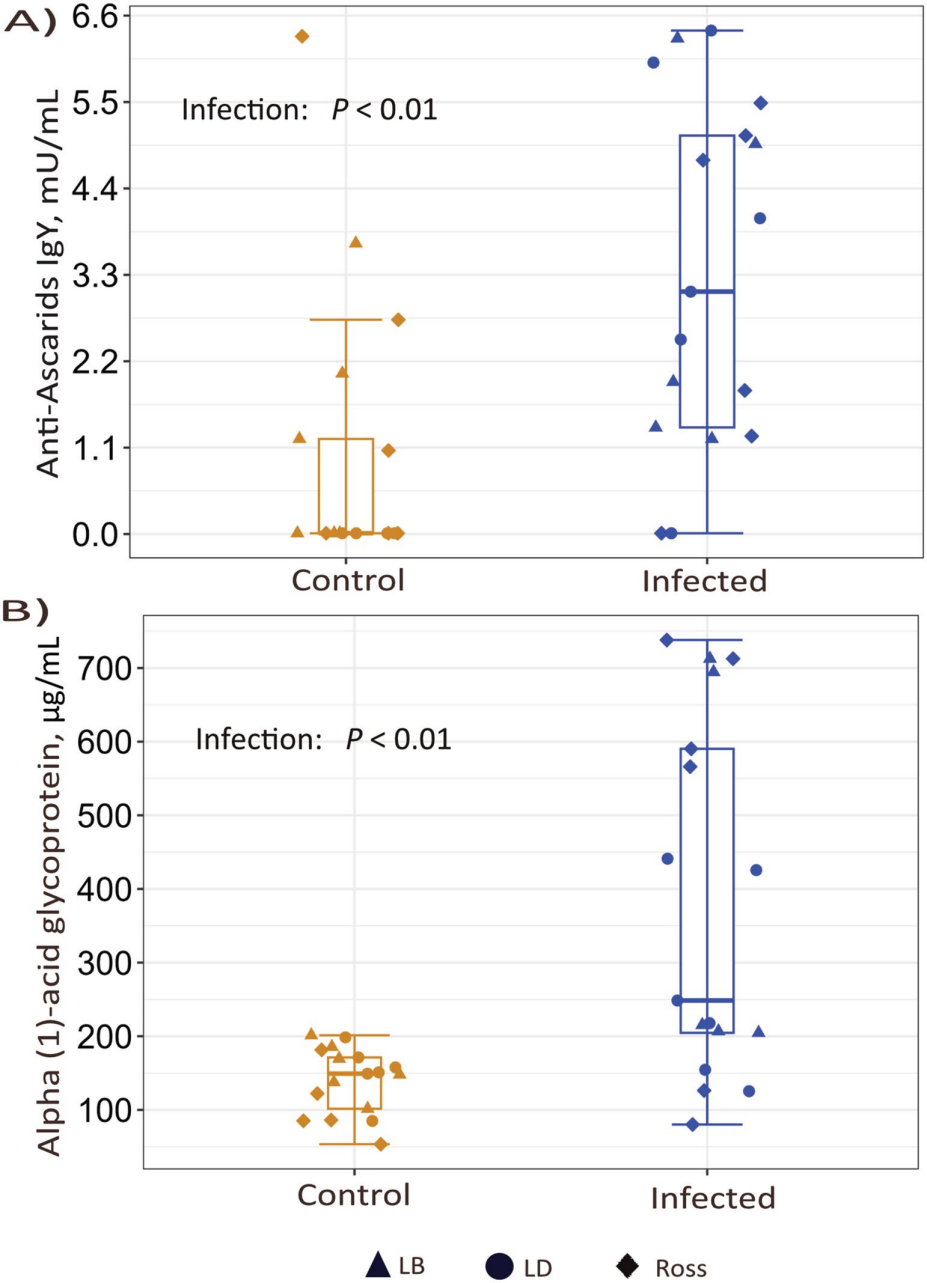
Box plots depicting **A** plasma anti-ascarid IgY concentration and **B** alpha-1-acid glycoprotein concentrations in infected (blue) and control (orange) chickens. Each dot represents an individual data point. The box plots display the median (central horizontal line), interquartile range (IQR, box edges representing the 25th and 75th percentiles). Error bars represent the standard error of the mean (SEM)

All birds inoculated with parasite eggs were confirmed as positively infected with both nematodes during necropsy. Ascarid-specific antibody levels were significantly higher in infected birds compared to the control group (*p* < 0.01), with no significant differences among strains. Similarly, alpha-1-acid glycoprotein levels were significantly elevated in infected birds (*p* < 0.01) (Fig. 1).

**Fig. 2:**
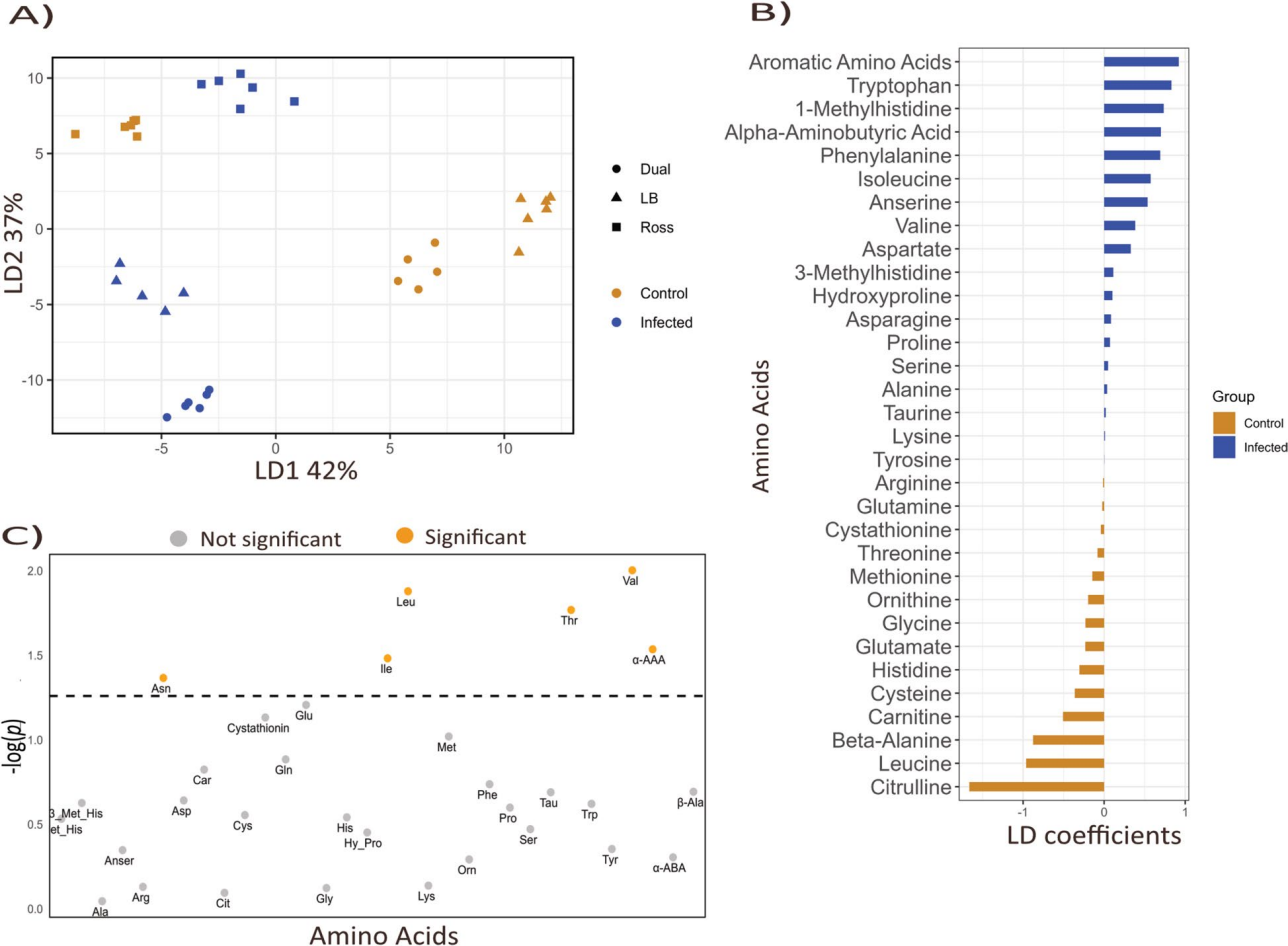
Plasma amino acid profiles. **A** Linear Discriminant Analysis (LDA) plot illustrating the separation of infection and strain groups based on amino acid profiles. Groups are color-coded by infection status, while different shapes represent the chicken strains. LD1 and LD2 capture the most variance in the dataset, highlighting distinct signatures between groups. **B** Loading bar plot showing the contribution of individual amino acids to the differentiation between infected (blue) and control (orange) groups. Longer bars indicate amino acids with a stronger influence on group separation, while shorter bars represent weaker contributors. Amino acids positively associated with control are in orange, whereas those linked to infection are in blue. **C** Displays the significance in amino acid concentrations between infected and control groups. The y-axis represents the -log₁₀(*p*) andamino amino acids significantly altered at *p* < 0.05 are highlighted. Aspartic Acid (Asp, Aspartate), Glutamic Acid (Glu, Glutamate), Cysteine (Cys), Alpha-Aminoadipic Acid (α-AAA), Asparagine (Asn), Serine (Ser), Glutamine (Gln), Histidine (His), Glycine (Gly), Threonine (Thr), Citrulline (Cit), Beta-Alanine (β -Ala), Arginine (Arg), Alanine (Ala), Carnosine (Car), Taurine (Tau), Anserine (Anser), Tyrosine (Tyr), Alpha-Aminobutyric Acid (α-ABA), Cystathionine,Valine (Val), Methionine (Met), Tryptophan (Trp), Phenylalanine (Phe), Isoleucine (Ile), Ornithine (Orn), Leucine (Leu), Lysine (Lys), Hydroxyproline (Hy-Pro), and Proline (Pro)

A linear discriminant analysis (LDA) differentiated control and infected samples based on 33 amino acids (Fig. 2 A–C). Overall, infected and control groups clustered separately along LD1 and LD2. Within each infection group, samples further grouped by strain, except for Ross birds (Fig. 2A). Notably, alpha-aminoadipic acid (α-AAA) (*p* = 0.029) and valine (Val) (*p* = 0.010) had positive LD1 coefficients (Fig. 2B) and had significantly lower concentrations in the infected group (Fig. 2C). Similarly, isoleucine (Ile) and threonine (Thr) had moderate loadings and were also significantly reduced in infected birds (*p* < 0.05). In contrast, leucine (Leu) (*p* = 0.013) exhibited strongly negative loadings and were present in higher concentrations in the control group. Asparagine (Asn) was also significantly elevated in the control group (*p* = 0.043). Table S1 presents the performance profiles of the birds including average daily gain (ADG) feed intake (FI) and feed conversion ratio (FCR).

### Transcriptomic changes due to infection in three strains across three tissues

**Fig. 3:**
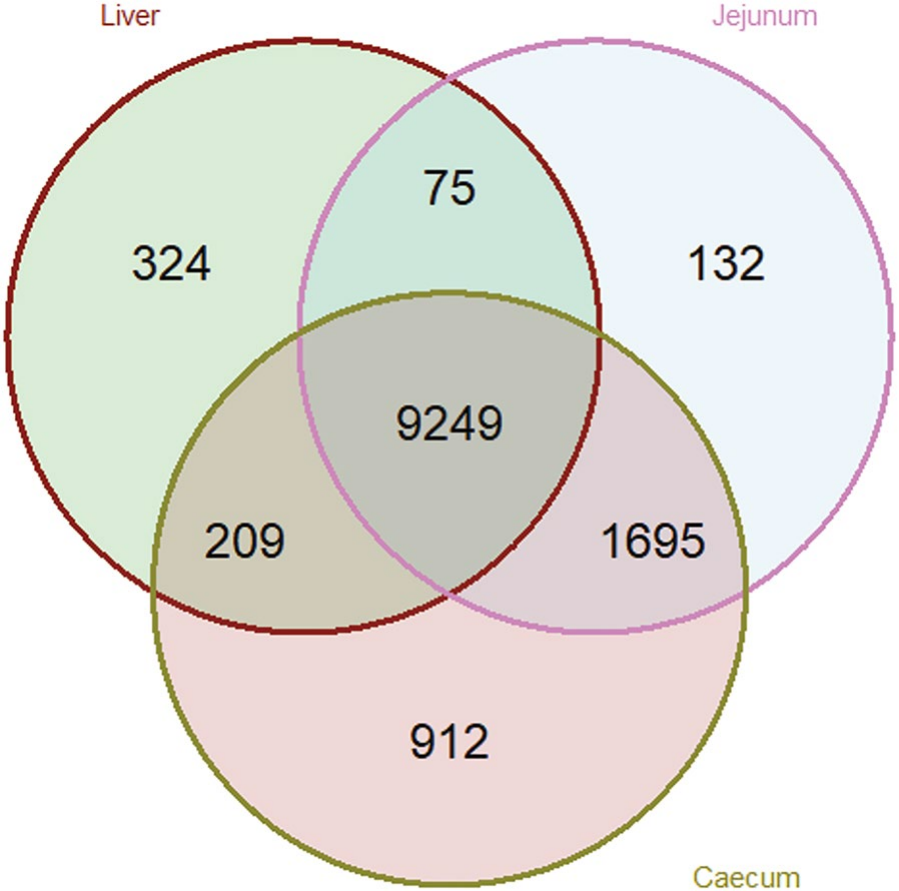
Venn diagram of expressed genes across tissues. Venn diagram illustrating the number of expressed genes in the liver jejunum, and caecum across infected vs control groups. Overlapping regions indicate genes commonly expressed in two or more tissues, while unique sections represent tissue-specific gene expression

**Fig. 4:**
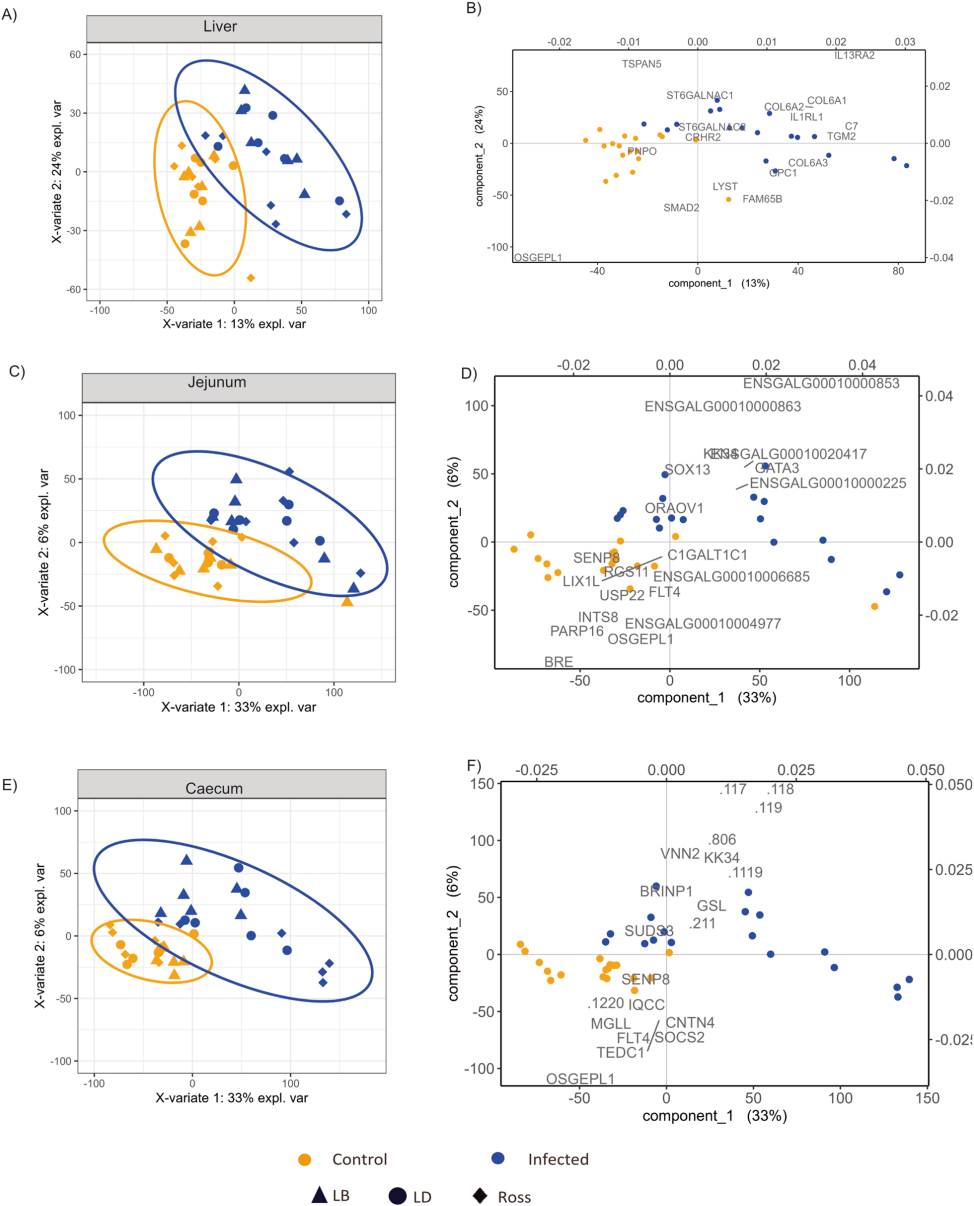
Sparse partial least squares discriminant analysis (sPLS-DA) of gene expression across tissues. sPLS-DA plots illustrating transcriptomic variation between infected and control groups in the liver, jejunum, and caecum. Each tissue is represented by: **A**, **C**, **E** Score plots of sPLS-DA for liver (**A**), jejunum (**C**), and caecum (**E**). Samples are coloured by infection status and shaped by strain, with 95% confidence ellipses indicating group clustering. The separation along the first two components reflects transcriptomic differences associated with infection and strain effects. **B**, **D**, **F** Corresponding biplots for each tissue (liver (**B**), jejunum (**D**), and caecum (**F**)), showing the contribution of key genes driving the separation between infected and control groups. Genes shown represent those with the strongest influence on the variance captured by Component 1 and Component 2. The direction and magnitude of these loadings highlight infection-associated transcriptional shifts within each tissue

Based on the RNA-Seq results, after filtering for genes with at least 100 counts in minimum of 5 samples, a total of 12,596 genes were found to be expressed across all three tissues. The number of expressed genes across the tissues is shown collectively for three strains in Fig. 3. We then performed sPLS-DA for each tissue to characterize the influence of infection status and strain on the overall transcriptomic profiles (Fig. 4). Infection status was the primary driver of sample separation across all tissues, whereas differentiation among strains was less pronounced. In the liver, the genes Transglutaminase 2 (*TGM2*), Complement Component 7 (*C7*), Collagen Type VI Alpha 1 Chain (*COL6A1*), and Interleukin 13 Receptor Subunit Alpha 2 (*IL13RA2*) strongly influenced the separation of infected samples, whereas Family with Sequence Similarity 65 Member B (*FAM65B*), Pyridoxine-5'-phosphate Oxidase (*PNPO*), and O-Sialoglycoprotein Endopeptidase-Like 1 (*OSGEPL1*) had maximum median in control samples. In the jejunum, the infected samples showed maximum median for the genes SRY-Box Transcription Factor 13 (*SOX13*) and GATA Binding Protein 3 (*GATA3*), while SUMO Peptidase Family Member 8 (*SENP8*), Brain and Reproductive Organ-Expressed (*BRE*), Fms-Related Tyrosine Kinase 4 (*FLT4*), and *OSGEPL1* were abundant in control samples. In the caecum, Vanin-2 (*VNN2*), Keratin 34 (*KK34*), Gibberellin Stimulated-Like proteins (*GSL)* and SIN3A Corepressor Complex Component *(SUDS 3*) were linked to infected samples, whereas OSGEPL1, SENP8, and Tubulin Epsilon and Delta Complex 1 (*TEDC1*) were more prominent in the control samples.

### Differentially expressed genes and enriched GO biological process terms in three tissues following parasite coinfections

**Fig. 5:**
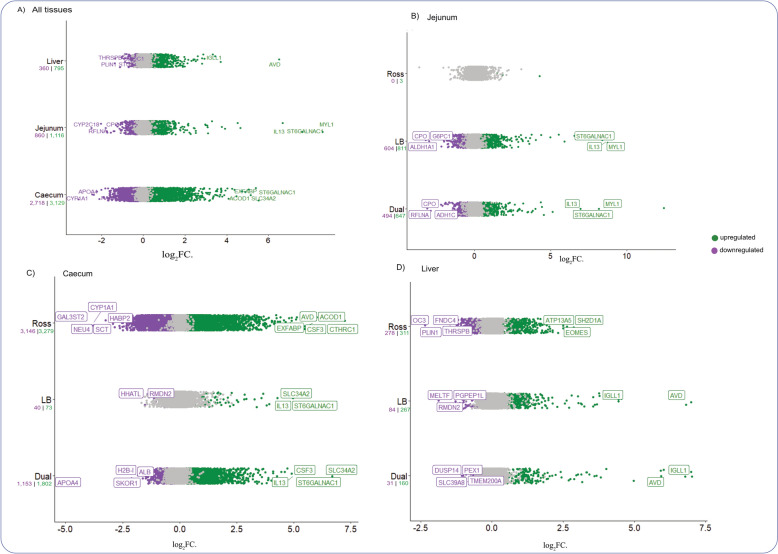
Volcano plots of differentially expressed genes (DEGs) in infected vs. control chickens across tissues and strains. Volcano plots illustrating differentially expressed genes (DEGs) between infected and control groups: **A** Overall DEGs across all tissues (jejunum, caecum, and liver). **B** DEGs in the jejunum, grouped by strain. **C** DEGs in the caecum, grouped by strain. **D** DEGs in the liver, grouped by strain. Each point represents a gene, with the x-axis showing log₂ fold change (log₂FC), indicating the expression direction in infected vs. control birds. Genes significantly upregulated in infected birds (FDR < 0.05) are shown in green, while those significantly downregulated in infected birds are in purple. Non-significant genes are displayed in grey

To characterize the effects of infection, we assessed the number of differentially expressed genes (DEGs) and the GO BP terms enriched by these genes across all tissues, with further stratification by strain. Among the tissues analysed, the caecum exhibited the most pronounced transcriptomic changes in response to the infections with *H. gallinarum* and *H. meleagridis*. In the caecum, 5,847 genes were differentially expressed, with 3,129 genes upregulated and 2,718 downregulated (FDR < 0.05) (Fig. 5A). In comparison, the liver and jejunum displayed 1,155 and 1,976 significantly DEGs, respectively. In the jejunum, 1,116 genes were upregulated and 860 downregulated, while in the liver, 795 genes were upregulated and 360 downregulated (Fig. 5A).

To assess the differences across strains, we examined the DEGs within birds of the same strain across tissues (Fig. 5). In the jejunum, only three genes were significantly upregulated in the Ross strain. In contrast, the jejunum of LB and LD birds showed 1,415 and 1,141 DEGs, respectively (Fig. 5B). In the liver and caecum, however, the Ross strain exhibited the most transcriptomic changes, with 589 DEGs in the liver (311 upregulated, 278 downregulated) and 6,425 (3,279 upregulated, 3,146 downregulated) in the caecum. In the liver, the LB and LD strains had 351 DEGs (267 upregulated, 84 downregulated) and 191 DEGs (160 upregulated, 31 downregulated), respectively. In the caecum, LB showed the fewest transcriptomic changes, with 113 DEGs (73 upregulated, 40 downregulated), while LD had 1,802 downregulated and 1,153 upregulated genes.

**Fig. 6:**
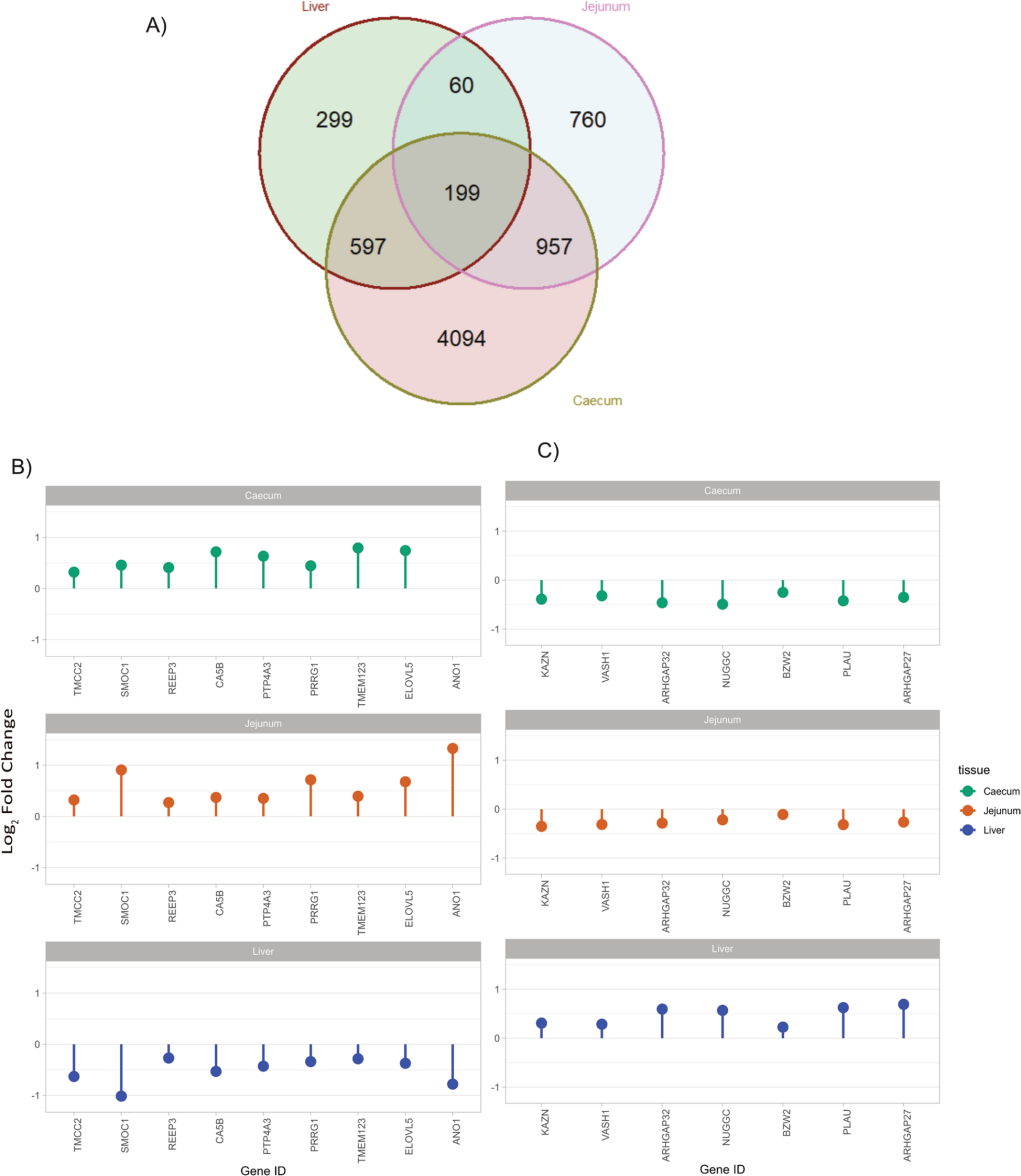
Shared and inversely regulated differentially expressed genes (DEGs) across tissues. **A** Venn diagram illustrating the overlap of differentially expressed genes (DEGs) across the jejunum, caecum, and liver, comparing infected vs. control birds. **B** Genes upregulated in the intestine (jejunum and caecum) but downregulated in the liver in response to infection. **C** Genes downregulated in the intestine (jejunum and caecum) but upregulated in the liver in response to infection

A comparison of gene expression profiles between infected and control groups across the three tissues revealed a substantial number of commonly regulated genes in more than one tissue, as well as tissue-specific genes (Fig. 6A). A total of 199 genes were differentially expressed in all three tissues. The jejunum and caecum shared the highest number of common DEGs, with 957 genes significantly expressed in both (FDR < 0.05). In contrast, only 60 DEGs were shared between the liver and jejunum, while 597 DEGs were common between the liver and caecum. The caecum exhibited the highest number of unique DEGs, with 4,094 genes not found in other tissues. In the liver, 299 DEGs were unique, whereas 760 DEGs were identified only in the jejunum. Across all three tissues, 253 significant genes were inversely regulated, meaning that genes downregulated in one tissue were upregulated in another. In Fig. 6B and C only the up- and down-regulated genes in the intestinal tissues (i.e. caecum and the jejunum) that were inversely regulated in the liver are shown.

**Fig. 7:**
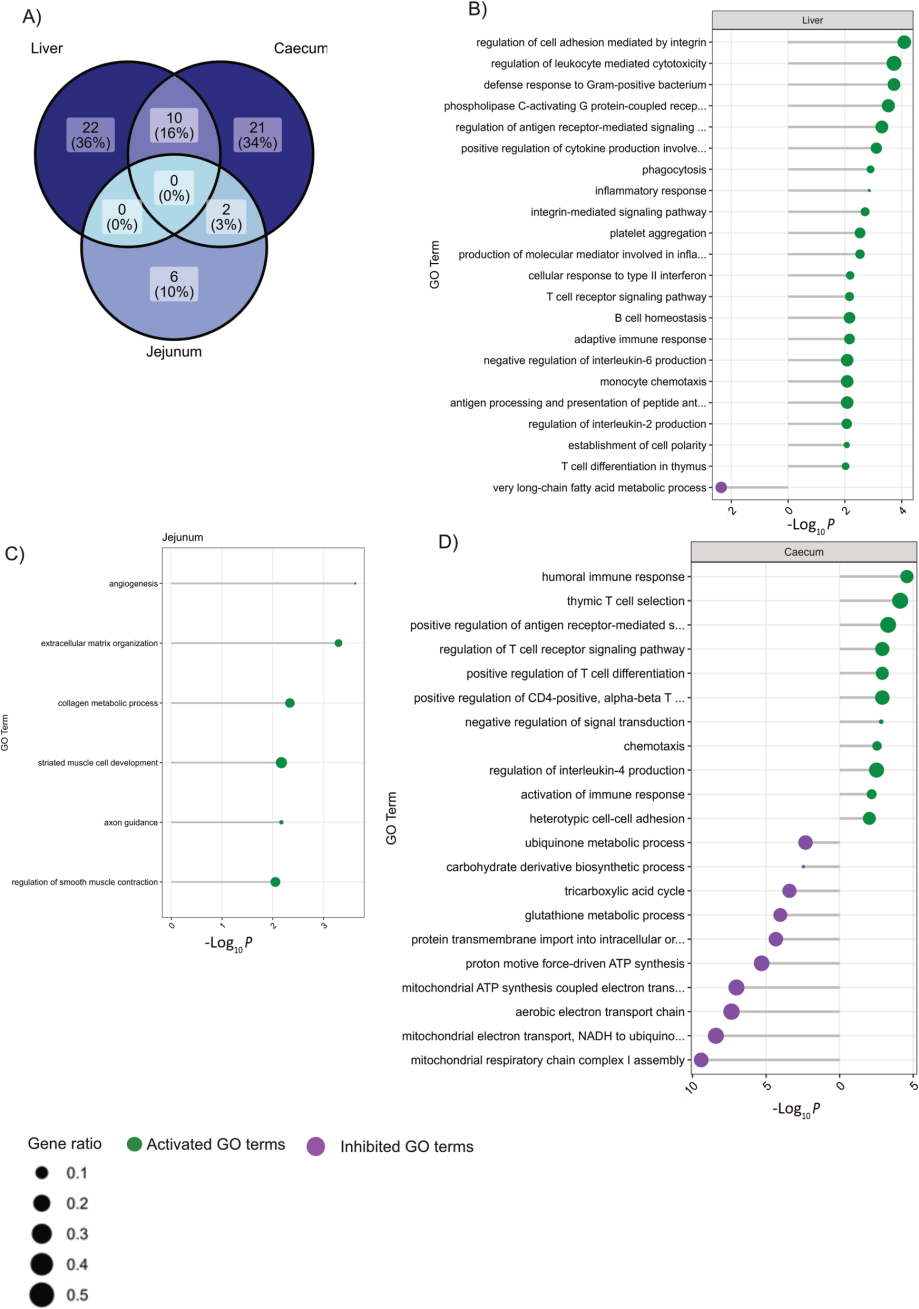
Significant biological process (BP) gene ontology (GO) terms across tissues. **A** Venn diagram showing the number and percentage of significant GO-BP (gene ontology-biological process) terms identified in the liver, jejunum, and caecum (adjusted *p* < 0.01), illustrating shared and tissue-specific enriched pathways. **B** GO-BP terms significantly enriched in the liver. **C** GO-BP terms significantly enriched in the jejunum. **D** GO-BP terms significantly enriched in the caecum. In panels **B**–**D**, green dots represent activated biological processes (enriched by genes upregulated in infected birds), while purple dots indicate inhibited biological processes (enriched by genes downregulated in infected birds). Dot size corresponds to the gene ratio (the proportion of observed significant genes relative to the expected number). The x-axis represents -log₁₀(adjusted *p*-value)

Gene ontology (GO) enrichment analysis was performed on the DEGs (FDR < 0.05) across all tissues combined, as well as for each tissue grouped by strain. The enriched GO terms (adj *p*-value < 0.01) selected are presented in Fig. 7 and Supplementary files S2 and S3.

**Fig. 8:**
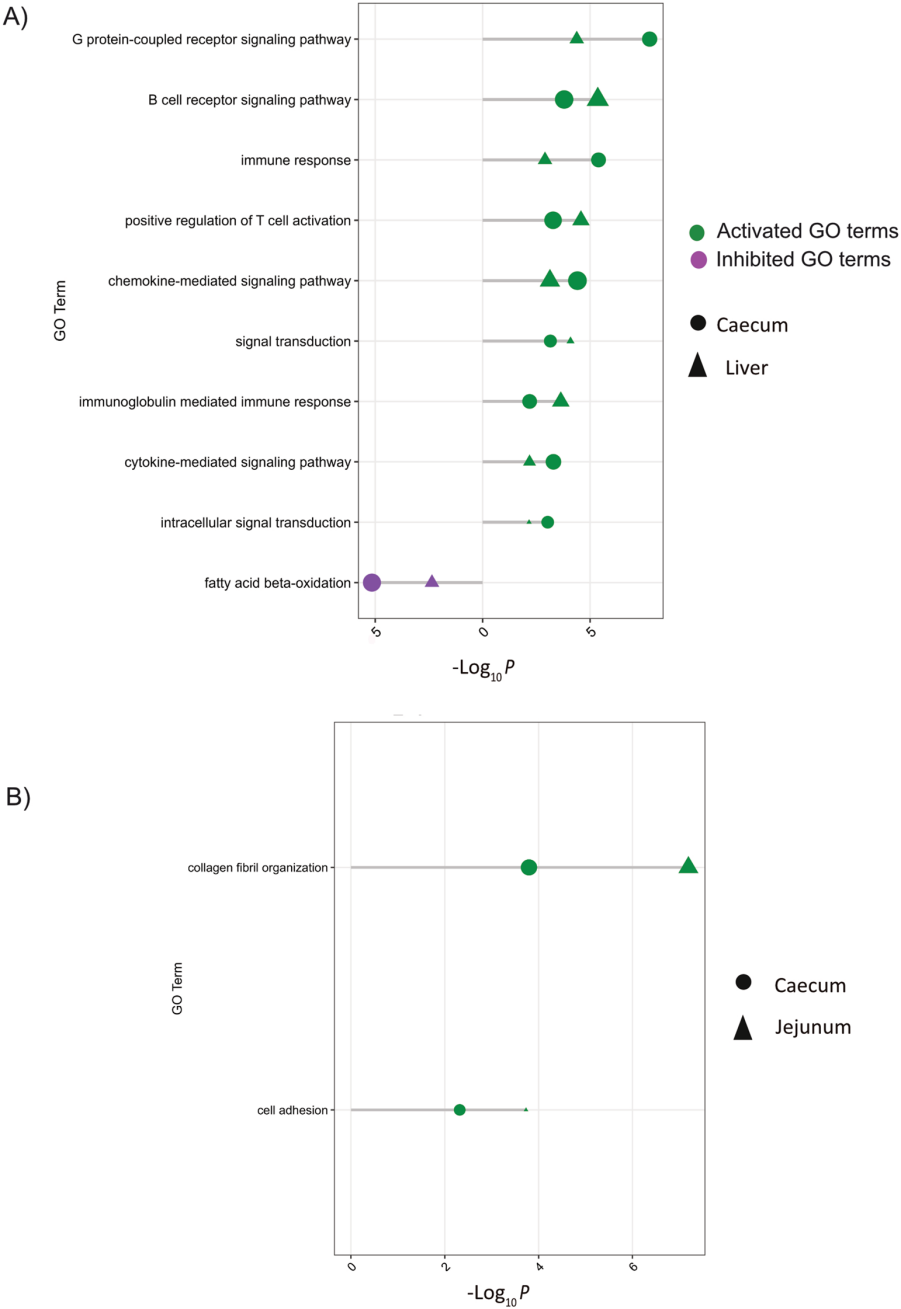
Significant gene ontology biological process (GO-BP) terms shared between tissues. **A** GO-BP terms significantly shared between the caecum and liver (adjusted *p* < 0.01). **B** GO-BP terms significantly shared between the caecum and jejunum (adjusted *p* < 0.01). Each panel highlights biological processes commonly enriched in the respective tissue pairs, emphasizing shared immune and metabolic responses to coinfection. Green dots indicate activated processes (enriched by upregulated genes), while purple dots represent inhibited processes (enriched by downregulated genes). Dot size corresponds to the gene ratio (proportion of observed significant genes relative to the expected number), and the x-axis represents − log₁₀(adjusted *p* -value)

The GO terms found in more than one tissue are shown in Fig. 8. There were 10 GO terms significantly enriched in both the liver and caecum (Fig. 8A), including fatty acid beta-oxidation (inhibited), cytokine-mediated signalling pathway, and immunoglobulin-mediated immune response (activated). Activation of collagen fibril organisation and cell adhesion were the common terms between caecum and jejunum (Fig. 8B). No significant GO terms were shared between liver and jejunum.

**Fig. 9:**
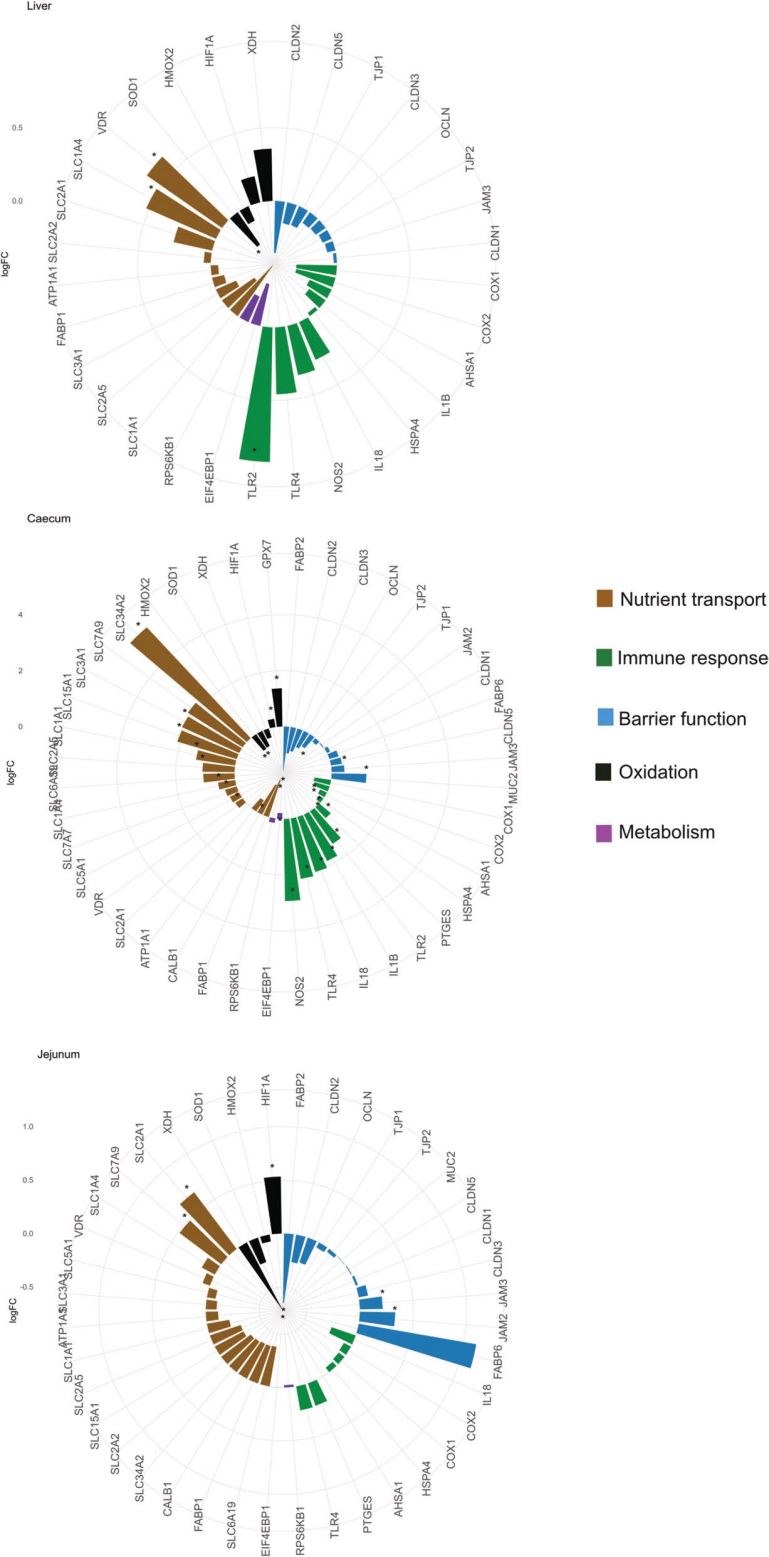
Circular bar plot of selected genes related to key biological functions. Circular bar plot illustrating log₂ fold change (log₂FC) of a custom-selected panel of genes associated with nutrient transport, immune response, barrier function, oxidation, and metabolism. Genes with significant differential expression (FDR < 0.05) are marked with an asterisk (*). Deep solid colours indicate upregulated genes, while lighter colours represent downregulated genes

Given the critical roles of barrier integrity, nutrient transport, metabolism, oxidation, and immune responses in shaping host defence mechanisms and overall physiological function, we specifically investigated the expression patterns of genes associated with these processes across the caecum, jejunum, and liver. As illustrated in Fig. 9, genes associated with barrier function displayed tissue-specific expression patterns. Barrier functions genes such as *CLDN1* (Claudin 1) and *JAM3* (Junctional Adhesion Molecule 3) were significantly upregulated in response to infection in both jejunum and caecum. In contrast, genes linked to nutrient transport, including *SLCs* (solute carrier family member) and *SLC5A1* (sodium/glucose cotransporter 1), exhibited significant downregulation in the jejunum and but significantly upregulated in the caecum and liver. Genes associated with immune responses showed a predominant upregulation in infected samples, with key pro-inflammatory mediators such as *IL1B* (Interleukin 1 Beta), *IL8* (Interleukin 8), displaying significant increases in expression across tissues. In contrast, genes involved in metabolism, including *EIF4EBP1* (Eukaryotic Translation Initiation Factor 4E Binding Protein 1), were downregulated in the liver.

## Discussion

In this study, we conducted a comparative analysis to explore how parasite coinfections transform the transcriptome across three key tissues—jejunum, caecum, and liver—representing predilection sites of three different parasites, to elucidate the tolerance mechanism in three different strains of chicken. Investigation of responses in chickens infected with nematodes have so far focused on mono-infections and thus examined the responses predominantly on single tissue [25, 29, 36] to understand the local or systemic responses in peripheral blood [20] or spleen [8]. A mono-parasite infection involves only one parasite species and is generally easier to study and interpret, while a mixed infection involves two or more parasite species-often with different biological traits, which can interact in complex ways. While mono-infections models provide insights into the overall immune responses to a specific parasite, they do not fully capture the complex pathophysiological responses occurring in coinfections as they occur under practical farming conditions. For instance, a mono *H. gallinarum* infection impairs body weight development of growing chickens, while an additional co-infection with *H. meleagridis* aggravates this effect on the host, but also reduces number of *H. gallinarum* [9]. In natural infections, where co-pathogens occupy distinct niches within the chicken host, the different local molecular responses in specific tissues are still unknown. As we have recently established [33], the performance level of the chicken strain also affects the extent of hepatic molecular responses to co-parasite infections. Therefore, in this study, we extended the investigation to two additional tissues (small intestine and caeca) to examine the responses in three chicken strains (LB, LD and Ross) with divergent performance levels, and gain insights into both tissue-specific and strain-dependent variations in host–pathogen interactions. Interestingly, we observed that infection altered the transcriptomic landscape of each tissue in an independent manner, revealing distinct tissue-specific responses to infection. The number of differentially expressed genes within each tissue provides an initial indication of tissue-specific responses. The caecum exhibited the highest number of significantly regulated genes among all tissues, with genes enriched in several GO terms related to immune responses (mostly activated) and metabolic processes (inhibited).

The caecum showed a pronounced activation of biological processes associated with the regulation of interleukin-4 (IL4) and IL2 production, and the positive regulation of CD4 + T cells. The concurrent activation of several immune pathways may be attributed to the presence of *H. meleagridis*, a protozoan transmitted via ingestion of *H. gallinarum* eggs, as the major vector [17]. Since the caecum is the primary predilection site for *H. gallinarum,* the entry *of H. gallinarum* allows the protozoa to invade the caecal wall using pseudopodia and elicit caecal responses before being transported to the liver likely via the hepatic portal vein system [49, 50]. The caecum, which serves as the first point of contact between the host and both *H. gallinarum* and *H. meleagridis*, may therefore account for the complex local immune response, particularly driven by T cell differentiation and increased cytokine production observed at 2 wpi. Although this time point reflects the effector phase of the adaptive response rather than the earliest innate events, it is plausible to speculate that strong local immune activation in the cecum contributes to limiting the translocation of *H. meleagridis* to the liver [37]. However, the LB strain—a strain with lowest growth rate in our study—appeared to show no inflammatory response to *H. meleagridis* and *H. gallinarum* in the caecum. In contrast, the high-performing Ross strain had the largest number of DEGs with a high impact on biological processes in the caecum. In general, several inhibited pathways, including ATP synthesis in mitochondria, the tricarboxylic acid (TCA) cycle, suggest that infection of the caecum may have compromised cellular function, including energy production pathways. The parasites likely induced oxidative stress—given the upregulation of Glutathione peroxidase 7 and Hypoxia-inducible factor 1-alpha— that impaired mitochondrial function and reduced the efficiency of ATP synthesis [3]. Protozoa like *Trypanosoma cruzi* infiltrating tissues of its mammalian host can directly decrease enzymatic activities of mitochondrial respiratory complexes, induce mitochondria dysfunction leading to oxidative stress [3, 14]. The significance of the inhibition of several mitochondrial functions in the caecum hosting histomonads is unknown and therefore warrants a study on the direct influence of parasites on mitochondrial function in avian hosts. This is also emphasized by the downregulation of the ubiquinone metabolic process, a mitochondrial cofactor that is crucial for electron transport in the mitochondria [15], which further supports the notion of an infection-related impairment of cellular energy state. In the jejunum, most upregulated biological processes are not directly linked to immune cell differentiation that are predominantly observed in the caecum. Instead, it reflects signalling adaptations evidenced by a strong myogenic alteration probably aimed at controlling intestinal barrier function, ‘weep and sweep’ response to worms or enhancing tissue resilience and repair. There exists in chicken a highly effective worm-expulsion mechanism demonstrating chickens’ ability to eliminate a significant proportion of parasitic worms through coordinated immune and physiological responses [45]. The activation of processes associated with smooth muscle contraction suggests a need for enhanced muscular support and contraction, an adaptive response likely to control gut motility, potentially to initiate the worm expulsion mechanisms [4]. The significant activation of collagen synthesis and muscle cell development pathways in the jejunum further suggests an active tissue remodelling and repair. Most importantly, these processes, which are associated with tissue remodelling and intestinal muscle contraction, were not evident in the Ross genotype, implying that the strains with slower growth rates, especially LB, more robustly regulate processes associated with tolerance and resilience.

Liver exhibited the lowest number of DEGs, even though it is well documented that the protozoan *H. meleagridis* migrates to the liver and causes necrotic lesions in infected hosts [50], which may explain increased AGP levels in the infected birds in line with the our previous results [32]. The reduced response by the liver compared to caecum could be because of the prominent early inflammatory, T-cell mediated response in the chicken gut which may have limited the spread of the protozoa to the liver [37]. Furthermore, our study was conducted only two weeks post-infection, a period during which the liver is unlikely to be highly attacked by histomonads. Nevertheless, a few of the protozoa likely made it to the liver which appears to have led to the activation of pathogen-directed cellular immunity observed in the liver tissue in the infected birds. Biological processes related to T cell-receptor signalling pathways, positive regulation of leukocyte-mediated cytotoxicity, antigen processing and presentation, and phagocytosis, were all activated, suggesting an active recruitment and engagement of both adaptive and innate immune systems. The host's phagocytic cells play a significant role in transporting the parasite. These cells pick up smaller forms of the parasite from the caeca and carry them through the portal system [49]. The immune response in the liver is likely an attempt to eliminate the parasite, target infected cells, or destroy cells presenting foreign antigens. It is intriguing to also observe an increase in the negative regulation of cytokine production in the liver. This could represent a homeostatic mechanism of the liver to prevent excessive immune activation and the risk of chronic inflammation and to enhance tolerance [18].

Fatty acid metabolism was significantly inhibited in the liver in line with our previous report obtained from another batch of infected animals [33]. The inhibition of this metabolic process can possibly be explained by the strategic shifting of liver function, prioritising immune-related processes and its homeostasis. The liver's attempt to maintain homeostasis during infection may come at a cost, potentially impairing its ability to meet the host's energy needs and contributing to reduced performance in infected animals. Although we did not observe a significant reduction in body weight gain due to infection in this cohort, reduced body weight gain is frequently associated with parasite infections. In the previous study with the same experimental setup, infection reduced weight gain and feed intake at subsequent weeks post infection [47]. Overall, while metabolic shift may give way for immediate immune responses, it could compromise long-term nutrient availability for strains under high performance pressure. Although this is the first study to provide a comprehensive transcriptomic analysis on tissue-specific responses to the mixed infection in chickens, certain limitations of this study should be acknowledged. A primary constraint is not quantifying the infectious dose of *H. meleagridis* administered to each bird, as the protozoan was delivered naturally via *H. gallinarum* eggs. While this method closely replicates natural infection dynamics typically observed in non-caged production systems, it may introduce variability in the actual exposure dose received by individual birds. An additional limitation lies in the absence of mono-infection groups, which precludes a clear distinction between parasite-specific and combined infection effects on host transcriptomic adaptations. Future research employing more controlled dosing strategies, alongside naturalistic models, would help to further clarify the effects observed and strengthen the conclusions drawn from such experimental designs.

## Conclusions

Our findings provide unique insight into the transcriptional adaptation induced by parasite coinfections in chicken’s caecum, liver, and jejunum, revealing tissue-specific oriented responses during the early phase of infections. Variations in response to coinfection were evident among strains particularly related to their performance levels.

The jejunum was dominated by the activation of biological processes related to tissue remodelling and muscle contraction, particularly in the slower-growing strains, suggesting a specific capacity for intestinal regeneration in birds under less production pressure. The caecum, which is the primary site of infection by *H. gallinarum* and the first host contact with *H. meleagridis* demonstrated a more complex and robust immune response. This enhanced response reflects the local immune activation and the stress of the simultaneous presence of parasites. Meanwhile, the liver maintained a more homeostatic, pathogen-targeted response, characterized by cytotoxic immune mechanisms aimed at the direct elimination of parasites. This response highlights the role of the liver in systemic immune regulation and its contribution to pathogen clearance.

Across all tissues, a clear prioritization of defence was observed. This metabolic reprogramming highlights the host's strategy of redistributing resources to the defence responses, possibly at the expense of growth and metabolic efficiency. Overall, these results improve our understanding of the complexity of immune and metabolic processes in chickens during parasite coinfections, with both tissue- and strain-dependent factors influencing the response to the coinfections.

## Supplementary Information

Supplementary file1

Supplementary Figure S2. Strain-Specific Significant Biological ProcessGene OntologyTerms Across Tissues.Significant GO-BP termsin the liver, by strain.Significant GO-BP termsin the jejunum, by strain.Significant GO-BP termsin the caecum, by strain. Each panel illustrates the biological processes enriched within each strain, highlighting strain-dependent immune and metabolic responses to coinfection. Activated biological processesare shown in green, while inhibited biological processesare shown in purple. The dot size represents the gene ratio, while the x-axis shows -log₁₀, indicating statistical significance.

Supplementary Table S3. Details of significant biological processgene ontologyterms across tissues and strains

## Data Availability

The Seq data was submitted to EMBL-EBI Annotare database with ArrayExpress accession number E-MTAB-14546. The RNA-seq and supporting data are available in the BioStudies databases (http://www.ebi.ac.uk/biostudies) under accession number E-MTAB-14546."Reviewer's share link: https://www.ebi.ac.uk/biostudies/arrayexpress/studies/E-MTAB-14546?key = 9222f656-5a4b-442b-b6a2-92bb48b3e0fd Further info: https://www.ebi.ac.uk/biostudies/about. Animal data and phenotypic measurements are deposited in ZENODO repository (DOI: https://doi.org/10.5281/zenodo.15052486).

## Abbreviations

**AGP**: Alpha (1)-acid glycoprotein
**BP**: Biological process
**DEG**: Differentially expressed genes
**FC**: Fold change
**FDR**: False discovery rate
**GO**: Gene ontology
**LB**: Lohmann Brown
**LD**: Lohmann Dual
**R**: Ross
**Th2**: T helper 2
**wpi**: Week post-infection
