# Supplementary material for: Tissue-specific transcriptomic adaptation in three strains of chickens during coinfections with parasites
Source: Gut Pathog. 2025 Jun 11;17:43. doi: 10.1186/s13099-025-00716-1 (PMC12160381; doi:10.1186/s13099-025-00716-1)
Supplement: Supplementary file 1 — Supplementary file1 [file 13099_2025_716_MOESM1_ESM.docx]

Table S1. Feed intake and growth performance in three strains of chickens co-infected with gastrointestinal nematodes and *Histomonas meleagridis*

|  | Infection | | |  | Strain | | | |  | Inf×Str |
| --- | --- | --- | --- | --- | --- | --- | --- | --- | --- | --- |
|  | Control | Infected | SE | Pval | Dual | LB | Ross | SE | Pval | Pval |
| ADF (g/day) | 37.7 | 36.1 | 1.68 | 0.518 | 25.65 | 18.59 | 66.10 | 2.06 | 0.001 | 0.904 |
| ADG (g/day) | 26.5 | 25.8 | 1.96 | 0.809 | 17.38 | 11.95 | 49.05 | 2.40 | 0.001 | 0.963 |
| FCR | 1.56 | 1.61 | 0.048871 | 0.475 | 1.60 | 1.66 | 1.48 | 0.06 | 0.001 | 0.520 |

ADF: Average Daily Feed (g/day); ADG: Average Daily Gain (g/day); FCR: Feed Conversion Ratio; Inf*Str: Infection × Strain interaction
