# Supplementary figures and images for: Tissue-specific transcriptomic adaptation in three strains of chickens during coinfections with parasites
Source: Gut Pathog. 2025 Jun 11;17:43. doi: 10.1186/s13099-025-00716-1 (PMC12160381; doi:10.1186/s13099-025-00716-1)

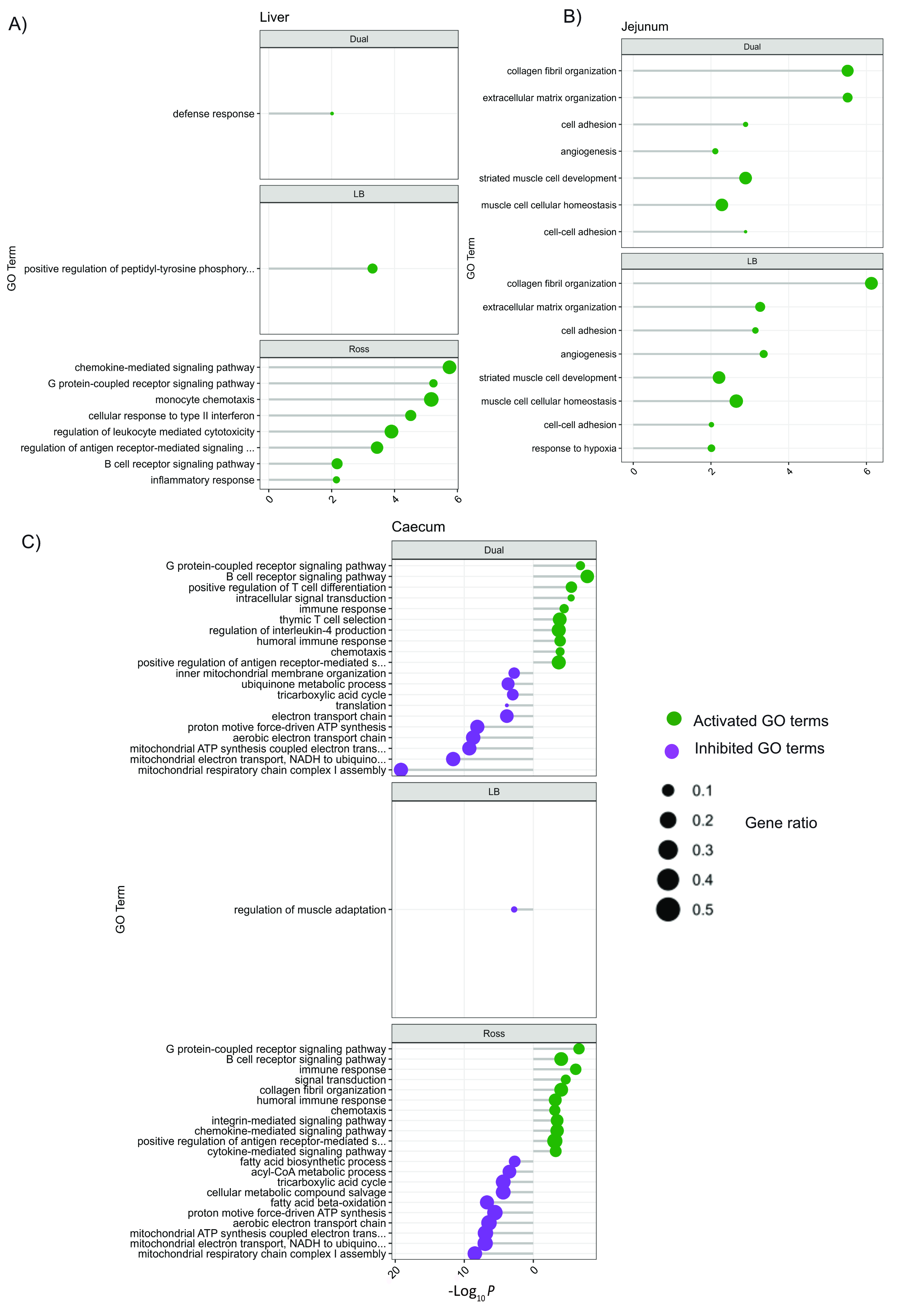

Supplement: Supplementary file 2 — Supplementary Figure S2. Strain-Specific Significant Biological ProcessGene OntologyTerms Across Tissues.Significant GO-BP termsin the liver, by strain.Significant GO-BP termsin the jejunum, by strain.Significant GO-BP termsin the caecum, by strain. Each panel illustrates the biological processes enriched within each strain, highlighting strain-dependent immune and metabolic responses to coinfection. Activated biological processesare shown in green, while inhibited biological processesare shown in purple. The dot size represents the gene ratio, while the x-axis shows -log₁₀, indicating statistical significance. [file 13099_2025_716_MOESM2_ESM.jpg]
