# Supplementary material for: Tissue-specific transcriptomic adaptation in three strains of chickens during coinfections with parasites
Source: Gut Pathog. 2025 Jun 11;17:43. doi: 10.1186/s13099-025-00716-1 (PMC12160381; doi:10.1186/s13099-025-00716-1)
Supplement: Supplementary file 3 — Supplementary Table S3. Details of significant biological processgene ontologyterms across tissues and strains [file 13099_2025_716_MOESM3_ESM.docx]

| **GO.ID** | **Term** | **Annotated** | **Significant** | **Expected** | **pvalue** | **adj_pvalue** | **regulation** | **tissue** | **Strain** |
| --- | --- | --- | --- | --- | --- | --- | --- | --- | --- |
| GO:0050731 | positive regulation of peptidyl-tyrosine phosphory... | 47 | 10 | 1.16 | 1.6e-07 | 0.00050016 | Activated | Liver | LB |
| GO:0006952 | defense response | 548 | 25 | 7.97 | 3.1e-06 | 0.0096906 | Activated | Liver | Dual |
| GO:0070098 | chemokine-mediated signaling pathway | 22 | 10 | 0.72 | 5.7e-10 | 1.78182E-06 | Activated | Liver | Ross |
| GO:0007186 | G protein-coupled receptor signaling pathway | 203 | 28 | 6.68 | 3.7e-09 | 5.7831E-06 | Activated | Liver | Ross |
| GO:0002548 | monocyte chemotaxis | 15 | 8 | 0.49 | 6.5e-09 | 0.000006773 | Activated | Liver | Ross |
| GO:0071346 | cellular response to type II interferon | 40 | 11 | 1.32 | 3.9e-08 | 3.04785E-05 | Activated | Liver | Ross |
| GO:0001910 | regulation of leukocyte mediated cytotoxicity | 15 | 7 | 0.49 | 2.0e-07 | 0.00012504 | Activated | Liver | Ross |
| GO:0050854 | regulation of antigen receptor-mediated signaling ... | 25 | 9 | 0.82 | 6.9e-07 | 0.00035949 | Activated | Liver | Ross |
| GO:0050853 | B cell receptor signaling pathway | 26 | 7 | 0.86 | 1.5e-05 | 0.006698571 | Activated | Liver | Ross |
| GO:0006954 | inflammatory response | 238 | 23 | 7.83 | 1.8e-05 | 0.0070335 | Activated | Liver | Ross |
| GO:0030199 | collagen fibril organization | 33 | 16 | 2.43 | 2.2e-10 | 7.436E-07 | Activated | Jejunum | LB |
| GO:0001525 | angiogenesis | 259 | 54 | 19.06 | 2.6e-07 | 0.0004394 | Activated | Jejunum | LB |
| GO:0030198 | extracellular matrix organization | 141 | 42 | 10.37 | 4.8e-07 | 0.0005408 | Activated | Jejunum | LB |
| GO:0007155 | cell adhesion | 582 | 92 | 42.82 | 8.5e-07 | 0.00071825 | Activated | Jejunum | LB |
| GO:0046716 | muscle cell cellular homeostasis | 15 | 8 | 1.1 | 3.3e-06 | 0.0022308 | Activated | Jejunum | LB |
| GO:0055002 | striated muscle cell development | 17 | 8 | 1.25 | 1.1e-05 | 0.006196667 | Activated | Jejunum | LB |
| GO:0001666 | response to hypoxia | 99 | 19 | 7.28 | 2.1e-05 | 0.0097175 | Activated | Jejunum | LB |
| GO:0098609 | cell-cell adhesion | 348 | 47 | 25.61 | 2.3e-05 | 0.0097175 | Activated | Jejunum | LB |
| GO:0030199 | collagen fibril organization | 33 | 14 | 1.92 | 1.3e-09 | 0.000003042 | Activated | Jejunum | Dual |
| GO:0030198 | extracellular matrix organization | 141 | 41 | 8.22 | 1.8e-09 | 0.000003042 | Activated | Jejunum | Dual |
| GO:0007155 | cell adhesion | 582 | 77 | 33.92 | 1.4e-06 | 0.0012844 | Activated | Jejunum | Dual |
| GO:0098609 | cell-cell adhesion | 348 | 41 | 20.28 | 1.7e-06 | 0.0012844 | Activated | Jejunum | Dual |
| GO:0055002 | striated muscle cell development | 17 | 8 | 0.99 | 1.9e-06 | 0.0012844 | Activated | Jejunum | Dual |
| GO:0046716 | muscle cell cellular homeostasis | 15 | 7 | 0.87 | 9.3e-06 | 0.005239 | Activated | Jejunum | Dual |
| GO:0001525 | angiogenesis | 259 | 39 | 15.09 | 1.6e-05 | 0.007725714 | Activated | Jejunum | Dual |
| GO:0043502 | regulation of muscle adaptation | 21 | 4 | 0.07 | 4.8e-07 | 0.0016872 | Deactivated | Caecum | LB |
| GO:0050853 | B cell receptor signaling pathway | 34 | 23 | 5.06 | 4.3e-12 | 1.51145E-08 | Activated | Caecum | Dual |
| GO:0007186 | G protein-coupled receptor signaling pathway | 314 | 98 | 46.74 | 8.3e-11 | 1.45873E-07 | Activated | Caecum | Dual |
| GO:0045582 | positive regulation of T cell differentiation | 44 | 21 | 6.55 | 2.6e-09 | 3.04633E-06 | Activated | Caecum | Dual |
| GO:0035556 | intracellular signal transduction | 1420 | 310 | 211.39 | 3.8e-09 | 3.33925E-06 | Activated | Caecum | Dual |
| GO:0006955 | immune response | 634 | 204 | 94.38 | 4.9e-08 | 0.000034447 | Activated | Caecum | Dual |
| GO:0006959 | humoral immune response | 44 | 21 | 6.55 | 2.3e-07 | 0.000130557 | Activated | Caecum | Dual |
| GO:0006935 | chemotaxis | 254 | 77 | 37.81 | 2.6e-07 | 0.000130557 | Activated | Caecum | Dual |
| GO:0045061 | thymic T cell selection | 17 | 12 | 2.53 | 3.4e-07 | 0.000149388 | Activated | Caecum | Dual |
| GO:0032673 | regulation of interleukin-4 production | 15 | 11 | 2.23 | 5.9e-07 | 0.000207385 | Activated | Caecum | Dual |
| GO:0050857 | positive regulation of antigen receptor-mediated s... | 15 | 11 | 2.23 | 5.9e-07 | 0.000207385 | Activated | Caecum | Dual |
| GO:0070098 | chemokine-mediated signaling pathway | 36 | 18 | 5.36 | 7.2e-07 | 0.000213829 | Activated | Caecum | Dual |
| GO:0019221 | cytokine-mediated signaling pathway | 202 | 66 | 30.07 | 7.3e-07 | 0.000213829 | Activated | Caecum | Dual |
| GO:0030890 | positive regulation of B cell proliferation | 24 | 14 | 3.57 | 1.1e-06 | 0.000297423 | Activated | Caecum | Dual |
| GO:0042102 | positive regulation of T cell proliferation | 37 | 18 | 5.51 | 1.2e-06 | 0.000301286 | Activated | Caecum | Dual |
| GO:0007165 | signal transduction | 2653 | 574 | 394.94 | 1.5e-06 | 0.0003515 | Activated | Caecum | Dual |
| GO:0050672 | negative regulation of lymphocyte proliferation | 38 | 15 | 5.66 | 1.6e-06 | 0.0003515 | Activated | Caecum | Dual |
| GO:0050856 | regulation of T cell receptor signaling pathway | 22 | 13 | 3.28 | 2.2e-06 | 0.000454882 | Activated | Caecum | Dual |
| GO:0019722 | calcium-mediated signaling | 94 | 36 | 13.99 | 2.6e-06 | 0.000507722 | Activated | Caecum | Dual |
| GO:0050852 | T cell receptor signaling pathway | 69 | 33 | 10.27 | 3.8e-06 | 0.000703 | Activated | Caecum | Dual |
| GO:0032722 | positive regulation of chemokine production | 26 | 14 | 3.87 | 4.1e-06 | 0.000703 | Activated | Caecum | Dual |
| GO:0032743 | positive regulation of interleukin-2 production | 23 | 13 | 3.42 | 4.4e-06 | 0.000703 | Activated | Caecum | Dual |
| GO:0046640 | regulation of alpha-beta T cell proliferation | 20 | 12 | 2.98 | 4.4e-06 | 0.000703 | Activated | Caecum | Dual |
| GO:0050729 | positive regulation of inflammatory response | 41 | 18 | 6.1 | 7.6e-06 | 0.001161478 | Activated | Caecum | Dual |
| GO:0030155 | regulation of cell adhesion | 376 | 123 | 55.97 | 3.3e-05 | 0.004833125 | Activated | Caecum | Dual |
| GO:0002768 | immune response-regulating cell surface receptor s... | 133 | 71 | 19.8 | 6.1e-05 | 0.008331852 | Activated | Caecum | Dual |
| GO:0030888 | regulation of B cell proliferation | 36 | 22 | 5.36 | 6.3e-05 | 0.008331852 | Activated | Caecum | Dual |
| GO:0043368 | positive T cell selection | 21 | 11 | 3.13 | 6.4e-05 | 0.008331852 | Activated | Caecum | Dual |
| GO:0032729 | positive regulation of type II interferon producti... | 28 | 13 | 4.17 | 7.0e-05 | 0.008504032 | Activated | Caecum | Dual |
| GO:0045577 | regulation of B cell differentiation | 18 | 10 | 2.68 | 7.2e-05 | 0.008504032 | Activated | Caecum | Dual |
| GO:0006954 | inflammatory response | 302 | 93 | 44.96 | 7.3e-05 | 0.008504032 | Activated | Caecum | Dual |
| GO:0042531 | positive regulation of tyrosine phosphorylation of... | 15 | 9 | 2.23 | 7.5e-05 | 0.008504032 | Activated | Caecum | Dual |
| GO:0008277 | regulation of G protein-coupled receptor signaling... | 44 | 14 | 6.55 | 8.9e-05 | 0.009776094 | Activated | Caecum | Dual |
| GO:0032981 | mitochondrial respiratory chain complex I assembly | 43 | 32 | 4.37 | 1.9e-23 | 6.6785E-20 | Deactivated | Caecum | Dual |
| GO:0006120 | mitochondrial electron transport, NADH to ubiquino... | 26 | 20 | 2.64 | 1.4e-15 | 2.4605E-12 | Deactivated | Caecum | Dual |
| GO:0042775 | mitochondrial ATP synthesis coupled electron trans... | 49 | 37 | 4.98 | 4.6e-13 | 5.38967E-10 | Deactivated | Caecum | Dual |
| GO:0019646 | aerobic electron transport chain | 45 | 35 | 4.57 | 2.2e-12 | 1.93325E-09 | Deactivated | Caecum | Dual |
| GO:0015986 | proton motive force-driven ATP synthesis | 20 | 15 | 2.03 | 1.1e-11 | 7.733E-09 | Deactivated | Caecum | Dual |
| GO:0006412 | translation | 446 | 70 | 45.29 | 2.7e-07 | 0.000145621 | Deactivated | Caecum | Dual |
| GO:0022900 | electron transport chain | 77 | 53 | 7.82 | 2.9e-07 | 0.000145621 | Deactivated | Caecum | Dual |
| GO:0006743 | ubiquinone metabolic process | 16 | 10 | 1.62 | 5.0e-07 | 0.000219688 | Deactivated | Caecum | Dual |
| GO:0006099 | tricarboxylic acid cycle | 22 | 11 | 2.23 | 2.7e-06 | 0.0010545 | Deactivated | Caecum | Dual |
| GO:0007007 | inner mitochondrial membrane organization | 23 | 11 | 2.34 | 4.8e-06 | 0.0016872 | Deactivated | Caecum | Dual |
| GO:0033108 | mitochondrial respiratory chain complex assembly | 66 | 45 | 6.7 | 7.8e-06 | 0.002492455 | Deactivated | Caecum | Dual |
| GO:0006119 | oxidative phosphorylation | 73 | 48 | 7.41 | 1.7e-05 | 0.004979583 | Deactivated | Caecum | Dual |
| GO:1904951 | positive regulation of establishment of protein lo... | 148 | 19 | 15.03 | 2.5e-05 | 0.006759615 | Deactivated | Caecum | Dual |
| GO:0033617 | mitochondrial cytochrome c oxidase assembly | 15 | 8 | 1.52 | 3.7e-05 | 0.008670333 | Deactivated | Caecum | Dual |
| GO:0009220 | pyrimidine ribonucleotide biosynthetic process | 15 | 8 | 1.52 | 3.7e-05 | 0.008670333 | Deactivated | Caecum | Dual |
| GO:0007186 | G protein-coupled receptor signaling pathway | 314 | 146 | 84.63 | 6.9e-11 | 2.42535E-07 | Activated | Caecum | Ross |
| GO:0006955 | immune response | 634 | 303 | 170.87 | 4.1e-10 | 7.20575E-07 | Activated | Caecum | Ross |
| GO:0007165 | signal transduction | 2653 | 958 | 715.02 | 1.7e-08 | 1.99183E-05 | Activated | Caecum | Ross |
| GO:0030199 | collagen fibril organization | 34 | 24 | 9.16 | 1.3e-07 | 0.00009139 | Activated | Caecum | Ross |
| GO:0050853 | B cell receptor signaling pathway | 34 | 24 | 9.16 | 1.3e-07 | 0.00009139 | Activated | Caecum | Ross |
| GO:0007229 | integrin-mediated signaling pathway | 52 | 31 | 14.01 | 7.1e-07 | 0.000356521 | Activated | Caecum | Ross |
| GO:0070098 | chemokine-mediated signaling pathway | 36 | 24 | 9.7 | 7.1e-07 | 0.000356521 | Activated | Caecum | Ross |
| GO:0019221 | cytokine-mediated signaling pathway | 202 | 103 | 54.44 | 1.3e-06 | 0.000571188 | Activated | Caecum | Ross |
| GO:0006959 | humoral immune response | 44 | 27 | 11.86 | 1.7e-06 | 0.000663944 | Activated | Caecum | Ross |
| GO:0050857 | positive regulation of antigen receptor-mediated s... | 15 | 13 | 4.04 | 2.3e-06 | 0.000766909 | Activated | Caecum | Ross |
| GO:0006935 | chemotaxis | 254 | 115 | 68.46 | 2.4e-06 | 0.000766909 | Activated | Caecum | Ross |
| GO:0045061 | thymic T cell selection | 17 | 14 | 4.58 | 3.0e-06 | 0.000811154 | Activated | Caecum | Ross |
| GO:0050870 | positive regulation of T cell activation | 92 | 56 | 24.8 | 3.0e-06 | 0.000811154 | Activated | Caecum | Ross |
| GO:0007155 | cell adhesion | 624 | 259 | 168.18 | 8.5e-06 | 0.002067647 | Activated | Caecum | Ross |
| GO:0050856 | regulation of T cell receptor signaling pathway | 22 | 16 | 5.93 | 9.8e-06 | 0.002067647 | Activated | Caecum | Ross |
| GO:0045582 | positive regulation of T cell differentiation | 44 | 28 | 11.86 | 9.8e-06 | 0.002067647 | Activated | Caecum | Ross |
| GO:2000516 | positive regulation of CD4-positive, alpha-beta T ... | 20 | 15 | 5.39 | 1.0e-05 | 0.002067647 | Activated | Caecum | Ross |
| GO:0006954 | inflammatory response | 302 | 133 | 81.39 | 2.0e-05 | 0.003905556 | Activated | Caecum | Ross |
| GO:0032963 | collagen metabolic process | 42 | 27 | 11.32 | 2.3e-05 | 0.004255 | Activated | Caecum | Ross |
| GO:0048260 | positive regulation of receptor-mediated endocytos... | 21 | 15 | 5.66 | 2.7e-05 | 0.004633409 | Activated | Caecum | Ross |
| GO:0032673 | regulation of interleukin-4 production | 15 | 12 | 4.04 | 2.8e-05 | 0.004633409 | Activated | Caecum | Ross |
| GO:0032715 | negative regulation of interleukin-6 production | 17 | 13 | 4.58 | 2.9e-05 | 0.004633409 | Activated | Caecum | Ross |
| GO:0030335 | positive regulation of cell migration | 291 | 121 | 78.43 | 3.1e-05 | 0.004737609 | Activated | Caecum | Ross |
| GO:0002548 | monocyte chemotaxis | 24 | 16 | 6.47 | 5.4e-05 | 0.0078736 | Activated | Caecum | Ross |
| GO:0016064 | immunoglobulin mediated immune response | 49 | 22 | 13.21 | 5.6e-05 | 0.0078736 | Activated | Caecum | Ross |
| GO:0050778 | positive regulation of immune response | 310 | 146 | 83.55 | 6.6e-05 | 0.008922692 | Activated | Caecum | Ross |
| GO:0046640 | regulation of alpha-beta T cell proliferation | 20 | 14 | 5.39 | 7.2e-05 | 0.009289643 | Activated | Caecum | Ross |
| GO:0071346 | cellular response to type II interferon | 46 | 25 | 12.4 | 7.4e-05 | 0.009289643 | Activated | Caecum | Ross |
| GO:0032981 | mitochondrial respiratory chain complex I assembly | 43 | 34 | 11.45 | 9.6e-13 | 3.3744E-09 | Deactivated | Caecum | Ross |
| GO:0006120 | mitochondrial electron transport, NADH to ubiquino... | 26 | 23 | 6.92 | 6.0e-11 | 1.0545E-07 | Deactivated | Caecum | Ross |
| GO:0042775 | mitochondrial ATP synthesis coupled electron trans... | 49 | 44 | 13.04 | 9.9e-11 | 1.15995E-07 | Deactivated | Caecum | Ross |
| GO:0006635 | fatty acid beta-oxidation | 42 | 31 | 11.18 | 2.2e-10 | 1.93325E-07 | Deactivated | Caecum | Ross |
| GO:0019646 | aerobic electron transport chain | 45 | 41 | 11.98 | 5.4e-10 | 3.7962E-07 | Deactivated | Caecum | Ross |
| GO:0015986 | proton motive force-driven ATP synthesis | 20 | 18 | 5.32 | 4.6e-09 | 2.69483E-06 | Deactivated | Caecum | Ross |
| GO:0006099 | tricarboxylic acid cycle | 22 | 18 | 5.86 | 9.9e-08 | 4.34981E-05 | Deactivated | Caecum | Ross |
| GO:0043094 | cellular metabolic compound salvage | 22 | 18 | 5.86 | 9.9e-08 | 4.34981E-05 | Deactivated | Caecum | Ross |
| GO:0006637 | acyl-CoA metabolic process | 47 | 33 | 12.51 | 8.9e-07 | 0.000347594 | Deactivated | Caecum | Ross |
| GO:0006633 | fatty acid biosynthetic process | 70 | 35 | 18.64 | 5.7e-06 | 0.00200355 | Deactivated | Caecum | Ross |
| GO:0006119 | oxidative phosphorylation | 73 | 62 | 19.43 | 6.8e-06 | 0.002172909 | Deactivated | Caecum | Ross |
| GO:0044743 | protein transmembrane import into intracellular or... | 24 | 17 | 6.39 | 7.6e-06 | 0.002226167 | Deactivated | Caecum | Ross |
| GO:0007007 | inner mitochondrial membrane organization | 23 | 16 | 6.12 | 2.0e-05 | 0.005407692 | Deactivated | Caecum | Ross |
| GO:0006767 | water-soluble vitamin metabolic process | 28 | 18 | 7.45 | 3.2e-05 | 0.008034286 | Deactivated | Caecum | Ross |
